# GATA3 and APOBEC3B are prognostic markers in adrenocortical carcinoma and APOBEC3B is directly transcriptionally regulated by GATA3

**DOI:** 10.18632/oncotarget.27703

**Published:** 2020-09-08

**Authors:** Sudheer Kumar Gara, Monica Varun Tyagi, Dhaval Thakkur Patel, Kelli Gaskins, Justin Lack, Yi Liu, Electron Kebebew

**Affiliations:** ^1^Thoracic Surgery Branch, National Cancer Institute, National Institutes of Health, Bethesda, MD, USA; ^2^The Department of Surgery and Stanford Cancer Institute, Stanford University, Stanford, CA, USA; ^3^Department of Surgery, Medical College of Wisconsin, Milwaukee, WI, USA; ^4^Office of Science and Technology Resources, Frederick National Laboratory for Cancer Research, National Institutes of Health, Bethesda, MD, USA; ^5^Salubris Biotherapeutics, Gaithersburg, MD, USA; ^*^These authors contributed equally to this work & are first authors

**Keywords:** adrenocortical carcinoma, APOBEC3B, GATA3, prognosis, DNA damage

## Abstract

Recent evidence has implicated *APOBEC3B* (Apolipoprotein B mRNA editing enzyme catalytic subunit 3B) as a source of mutations in breast, bladder, cervical, lung, head, and neck cancers. However, the role of APOBEC3B in adrenocortical carcinoma (ACC) and the mechanisms through which its expression is regulated in cancer are not fully understood. Here, we report that APOBEC3B is overexpressed in ACC and it regulates cell proliferation by inducing S phase arrest. We show high *APOBEC3B* expression is associated with a higher copy number gain/loss at chromosome 4 and 8 and *TP53* mutation rate in ACC. GATA3 was identified as a positive regulator of *APOBEC3B* expression and directly binds the APOBEC3B promoter region. Both GATA3 and APOBEC3B expression levels were associated with patient survival. Our study provides novel insights into the function and regulation of APOBEC3B expression in addition to its known mutagenic ability.

## INTRODUCTION

Adrenocortical carcinoma (ACC) is a rare and aggressive endocrine malignancy [1, 2]. There have been significant advances in understanding the genetic alterations that are involved in ACC initiation and progression [3, 4]. Based on these genomic and genetic studies, molecular classification of ACC has been established, which has prognostic relevance.

Mutagenesis occurs from exogenous sources present in the environment and endogenous sources that are present intracellularly. Exogenous sources include ultraviolet radiation, that results in the transitions of cytosine to thymine (C-to-T) due to oxidative damage which eventually forms pyrimidine dimers, aristolochic acid, a dietary supplement derived from plants which is responsible for A-to-T transversion mutations in the liver and tobacco, an established carcinogen [5]. Endogenous sources can be categorized as active or passive. The inability to repair DNA damage is a characteristic of passive alteration, whereas active agents have a direct effect on DNA, for instance, hydrolytic deamination of methyl-cytosine as an age-related function [6].

The distinct pattern of DNA base alterations has been characterized in the cancer genome using high throughput deep sequencing technologies, that reflect the underlying mutational process. The kataegis pattern of hypermutation clusters, largely dominated by C-to-T transitions, had been identified as a hallmark of various cancers. Whole genome and exome mutation analysis of The Cancer Genome Atlas (TCGA) data on multiple cancers have revealed that this pattern is consistent with the deaminase activity of the AID/APOBEC (apolipoprotein B mRNA editing enzyme, catalytic polypeptide-like) family of enzymes, therefore, implying its significance as an endogenous mutator and a crucial contributor to somatic mutations and genomic instability [7, 8].

In humans, the AID/APOBEC family has eleven members including activation-induced cytidine deaminase (AID, gene name: AICDA) and apolipoprotein B mRNA editing enzymes, catalytic polypeptide-like (APOBECs): APOBEC1 (A1), APOBEC2 (A2), APOBEC3 (A3), APOBEC3A (A3A), APOBEC3B (A3B), APOBEC3C (A3C), APOBEC3DE (A3DE), APOBEC3F (A3F), APOBEC3G (A3G), APOBEC3H (A3H), and APOBEC4 (A4) [9]. The enzyme can specifically edit DNA or RNA by irreversible cytidine and deoxycytidine deamination, resulting in the conversion of target cytosine (C) to uracil (U), thereby causing DNA or RNA alterations/damages [10]. Investigators have shown the contribution of APOBEC3B in malignant transformation [11]. APOBEC3B is overexpressed in ovarian cancer cell lines and high-grade primary ovarian cancers. In addition, APOBEC3B expression is positively correlated with the total mutation load, as well as, elevated levels of transversion mutations [12].

Given there are no well-established exogenous factors associated with ACC, we postulated whether APOBEC3B could be an endogenous mechanism of genomic instability/mutations in ACC and investigated its function *in vitro* and *in vivo*.

## RESULTS

### *APOBEC3B* expression is upregulated in adrenocortical carcinoma (ACC)

**Figure 1:**
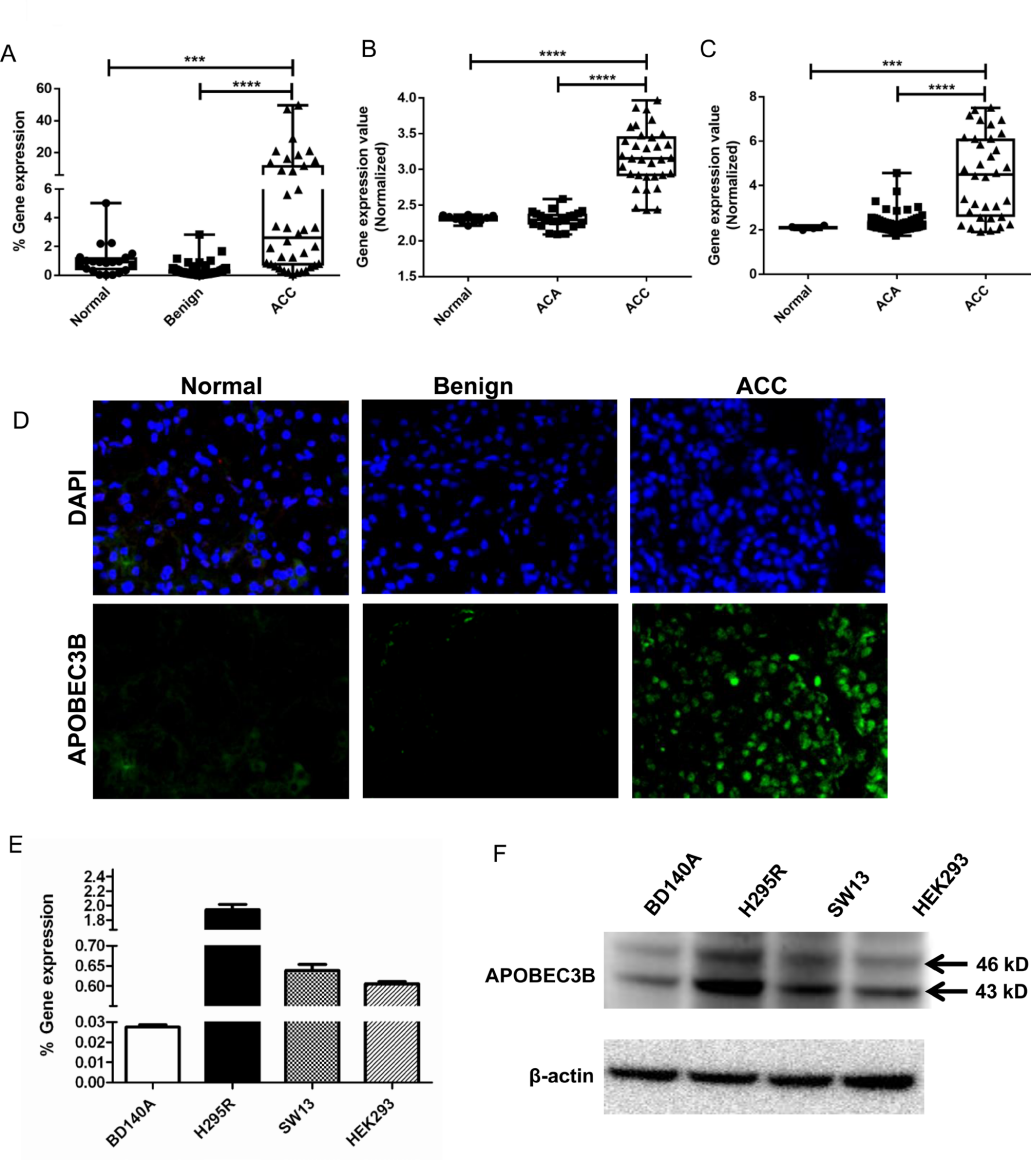
APOBEC3B expression in adrenocortical carcinoma. (**A**) TaqMan quantitative real-time PCR of APOBEC3B in ACCs in our cohort of adrenocortical tissue samples. Normal, *n* = 21; Benign, *n* = 69; ACC, *n* = 38. (**B**) APOBEC3B expression in publicly available microarray data from the GEO database, GSE10927. Normal, *n* = 10; ACA, *n* = 22; ACC, *n* = 33. The data were analyzed through GEO2R online software tool and all the data points were shown in Box-whiskers plots (**C**) APOBEC3B expression in another publicly available data from EMBL-EBI, E-TABM-311. Normal *n* = 4; ACA, *n* = 58; ACC, *n* = 34. ^*^ *P* < 0.05; ^**^ *P* < 0.01; ^***^ *P* < 0.001; ^****^ *P* < 0.0001. The author submitted normalized expression data were collected directly and all the data points were shown in Box-whiskers plots (**D**) Representative immunofluorescent staining images for APOBEC3B expression in adrenocortical tissues. DAPI is used to stain the cell nuclei for reference (magnification, 40×). TaqMan gene expression (**E**) and protein expression (**F**) of APOBEC3B in three adrenocortical carcinoma cell lines, BD140A, H295R, SW13, and normal human embryonic kidney cell line, HEK293. We used H295R and BD140 adrenocortical carcinoma cell lines for subsequent experiments as they are more representative of adrenocortical carcinomas.

We analyzed *APOBEC3B* gene expression in 21 normal adrenal cortices, 69 benign adrenocortical tumors and 38 primary ACC samples and found that APOBEC3B mRNA is significantly overexpressed in ACC (*p* < 0.001) (Figure 1A). We also analyzed two publicly available datasets from GEO (GSE10927 deposited by Giordano *et al*.) and EMBL-EBI (E-TABM-311 deposited by Reynies *et al*.) for the expression of APOBEC3B and observed that in both cohorts, there was a 5–6 fold higher expression in ACC compared to normal and benign adrenocortical tissue samples (*p* < 0.01) with no difference in expression between benign tumors and normal adrenocortical tissue samples (Figure 1B and 1C). APOBEC3B protein was also overexpressed, by immunofluorescence, in ACC while little or no expression was detected in normal adrenal cortex and benign adrenocortical tumors (Figure 1D). We evaluated APOBEC3B mRNA and protein expression in three adrenocortical cell lines (BD140, H295R, and SW13) and HEK293 cells, we detected two isoforms of APOBEC3B with a predicted molecular weight of ~43 and ~46 kD (Figure 1E and 1F). The RT-qPCR and Western blot analysis showed the highest level of APOBEC3B expression in the H295R cell line as compared to BD140A, SW13, and HEK293 cell lines. (Figure 1E and 1F, and Supplementary Figure 1). For subsequent experiements, we used H295R and BD140A cells as they are more representative of ACCs.

### Knockdown of APOBEC3B reduces cell proliferation and induces S phase cell cycle arrest

**Figure 2:**
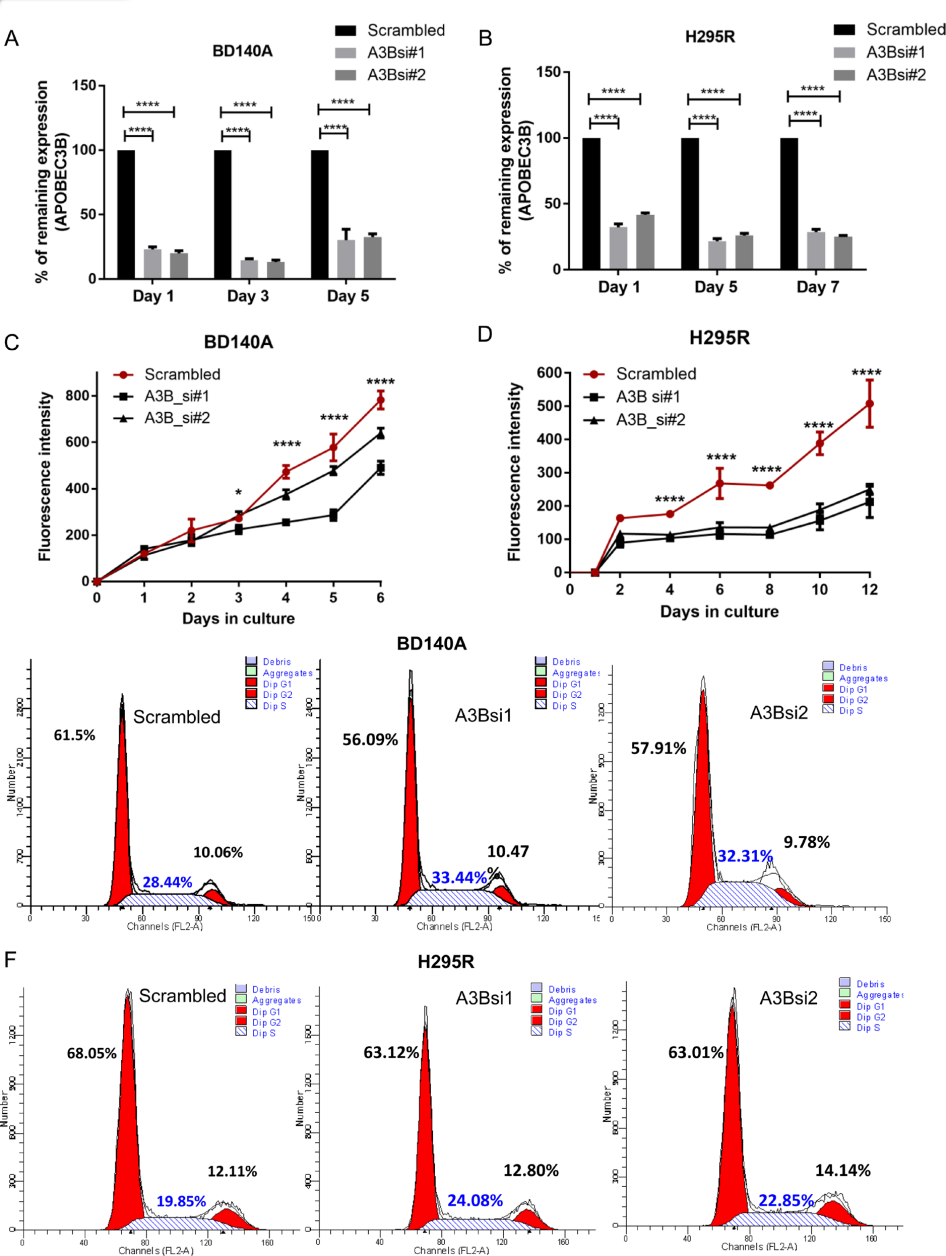
Knockdown of *APOBEC3B* reduces cell proliferation and induces S phase arrest. Knockdown of *APOBEC3B* gene expression over time using two siRNAs targeting (A3Bsi1, A3Bsi2) both isoforms in BD140A (**A**) and H295R (**B**) cell lines by TaqMan quantitative real-time PCR. Percentage expression has been normalized to scrambled siRNA which was used as a negative control. The cell proliferation growth curve after transient transfection of two siRNAs in BD140A (**C**) and H295R (**D**) cell lines as measured by CyQuant assay. ^*^ *P* < 0.05; ^***^ *P* < 0.001. Cell cycle analysis in BD140A (**E**) and H295R (**F**) cell lines after transient knockdown of APOBEC3B for 72 hours. The proportion of cells in each phase is represented as a percentage next to their corresponding peaks.

To assess the effect of APOBEC3B in cellular functions, we analyzed siRNA mediated transient knockdown of APOBEC3B gene expression with two different siRNAs in BD140A and H295R cell lines (Figure 2A and 2B). We achieved about 75–80% knockdown efficiency for 5 days and 9 days in BD140A and H295R cells, respectively, as validated by RT-qPCR and Western blotting (Figure 2A and 2B, and Supplementary Figure 2). We observed that reduced APOBEC3B gene expression significantly reduced cell proliferation in both cell lines (*p* < 0.001) (Figure 2C and 2D) and induced significant S-phase arrest (Figure 2D and 2F). However, we did not observe any significant difference in G1 or G2 cell cycle phases (Figure 2E and 2F, and Supplementary Figure 3).

### APOBEC3B is associated with double-strand breaks and higher TP53 mutation rate in ACC

**Figure 3:**
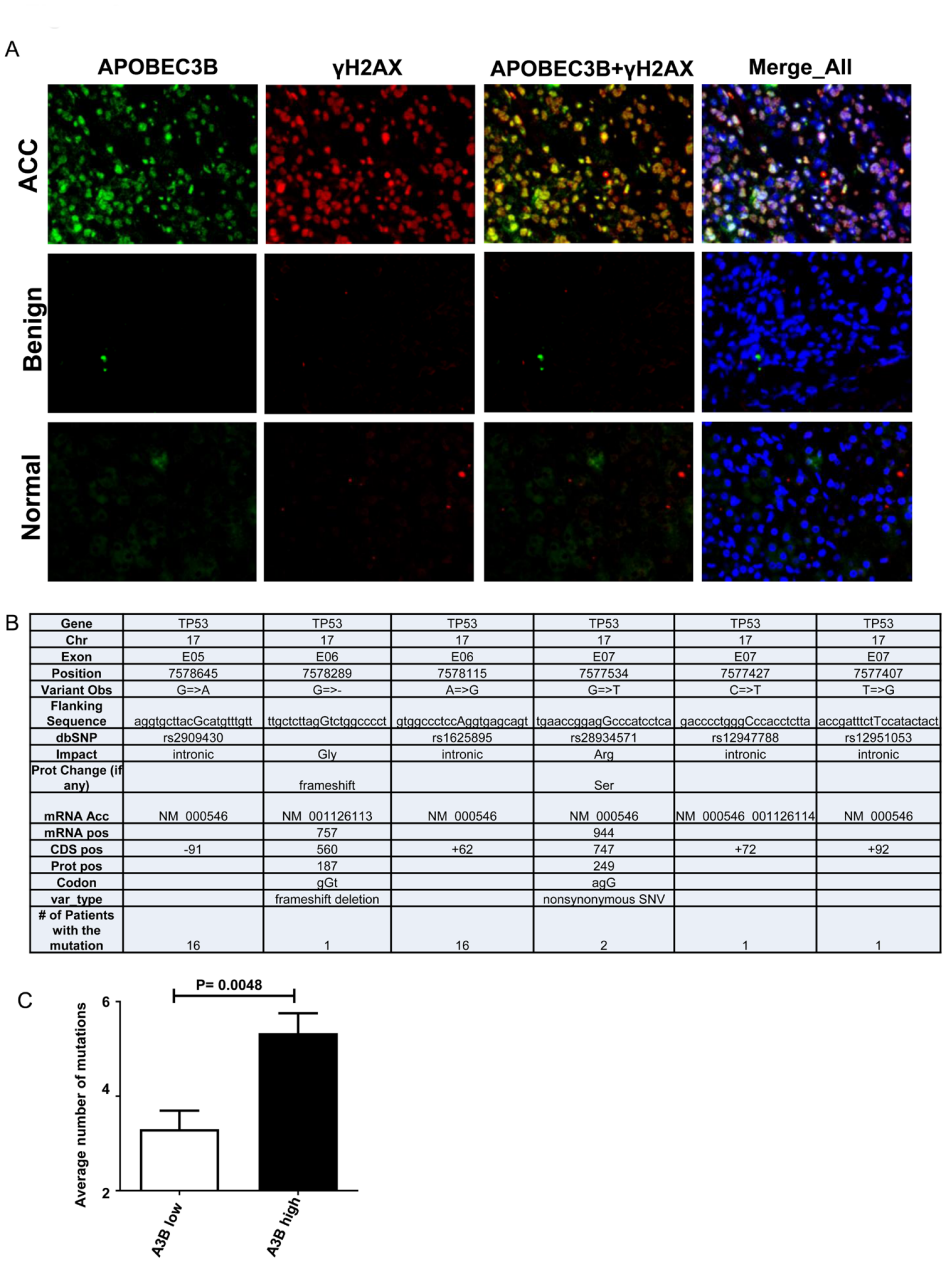
APOBEC3B is associated with DNA double stranded breaks and *TP53* mutations. (**A**) Representative co-immunofluorescent staining images of APOBEC3B and γH2AX in normal, benign adrenocortical tumors and adrenocortical carcinoma. DAPI was used to stain the cell nuclei as a reference. (magnification, 40×) (**B**) The table represents the list and type of mutations within the TP53 gene that was screened in adrenocortical tumors using Sanger DNA sequencing. (**C**) The adrenocortical tumors were stratified into two groups (high and low expression levels) based on the median gene expression level of APOBEC3B and the average number of observed *TP53* mutations in each patient was represented. The y-axis represents the sequencing data obtained from both forward and reverse strands for a given mutation. Error bar represents the standard error mean (SEM).

Since *APOBEC3B* is known to induce DNA damage, a critical molecular event in multiple cancers, we investigated the levels of phospho-Histone H2AX (Ser139), an indicator of DNA double-strand breaks. We observed high levels of γ-H2AX in ACC as compared to normal adrenal cortex and benign adrenocortical tumor samples (Figure 3A). The majority of phospho-H2AX staining was observed in the areas of tumor in six different tumor samples. Co-immunofluorescent staining analysis showed that APOBEC3B co-localizes with γH2AX within the nucleus of the ACC (Figure 3A and Supplementary Figure 4). However, there was only partial colocalization of γH2AX and APOBEC3B suggesting that APOBEC3B may only partially contribute to DNA double-strand breaks (Figure 3A and Supplementary Figure 4). Since *TP53* mutations are common in ACC, we analyzed if the expression level of APOBEC3B is associated with *TP53* gene mutations (Figure 3B). For this analysis, we dichotomized our ACC cohort based on the median gene expression level of APOBECB and performed Sanger sequencing for *TP53* gene mutations in the same tumor samples. We found that tumors with high APOBEC3B expression had twice the number of *TP53* mutations as compared to tumor samples with lower APOBEC3B expression (Figure 3C). Additionally, we observed a significant correlation between APOBEC3B gene expression level and the overall tumor burden in ACC tumor samples from the TCGA dataset (Supplementary Figure 5).

### A high APOBEC3B expression is associated with copy number loss and patient survival

**Figure 4:**
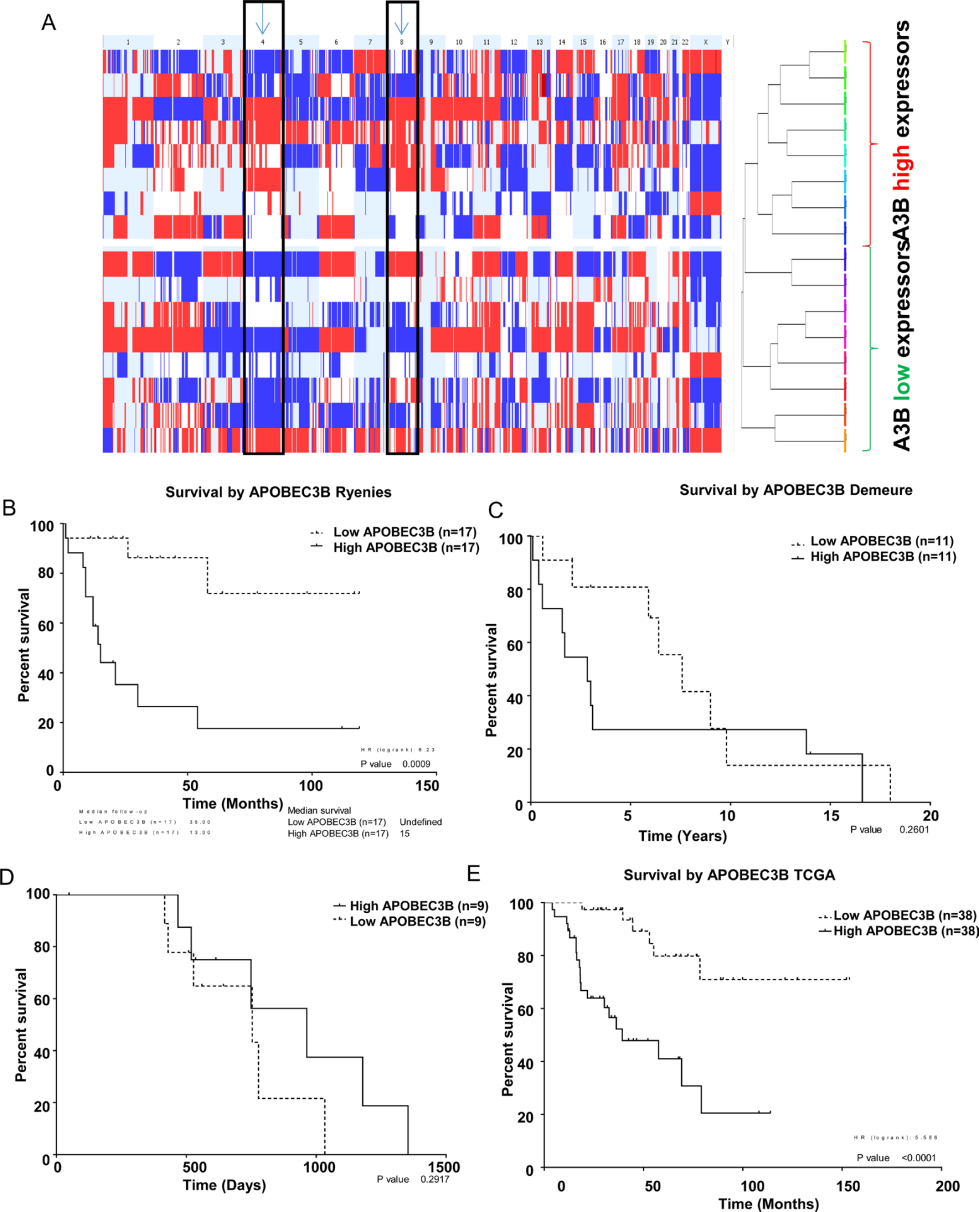
APOBEC3B expression is associated with copy number alterations. (**A**) Genome-wide CGH array representing cumulative copy number gain or loss for all chromosomes in adrenocortical carcinoma samples were dichotomized by the median gene expression level of APOBEC3B in the tumor samples. Red indicates the copy number loss while blue represents the copy number gain in a given chromosome. The columns represent each chromosome. Overall patient survival dichotomized based on the median gene expression level of APOBEC3B from two publicly available datasets; E-TABM-311 (**B**), GSE19776, (**C**) our cohort, (**D**) and the TCGA adrenocortical cancer data (**E**).

We performed a comparative genome hybridization (CGH) array in the ACC cohort and analyzed the association of APOBEC3B gene expression with gene copy number. We found that ACC with higher APOBEC3B gene expression had higher rates of chromosomal gain/loss particularly in chromosomes 4 and 8 as compared to samples with lower APOBEC3B expression (Figure 4A). Next, we analyzed whether APOBEC3B gene expression is associated with patient survival. We did not find any significant association between APOBEC3B gene expression and patient survival from two publicly available datasets (E-TABM-311 and GSE19776) and in our cohort (Figure 4B–4D). However, we found a significant association between APOBEC3B gene expression and patient overall survival and disease free survial in a larger cohort of ACC of The Cancer Genome Atlas (TCGA) study where patients with a higher level of APOBEC3B gene expression in their tumors had a lower survival rate as compared to patients with lower expression APOBEC3B gene expression in their tumors (Figure 4E and Supplementary Figure 6).

### APOBEC3B gene expression is regulated by GATA3 which binds the promoter region of APOBEC3B and is associated with patient survival

**Figure 5:**
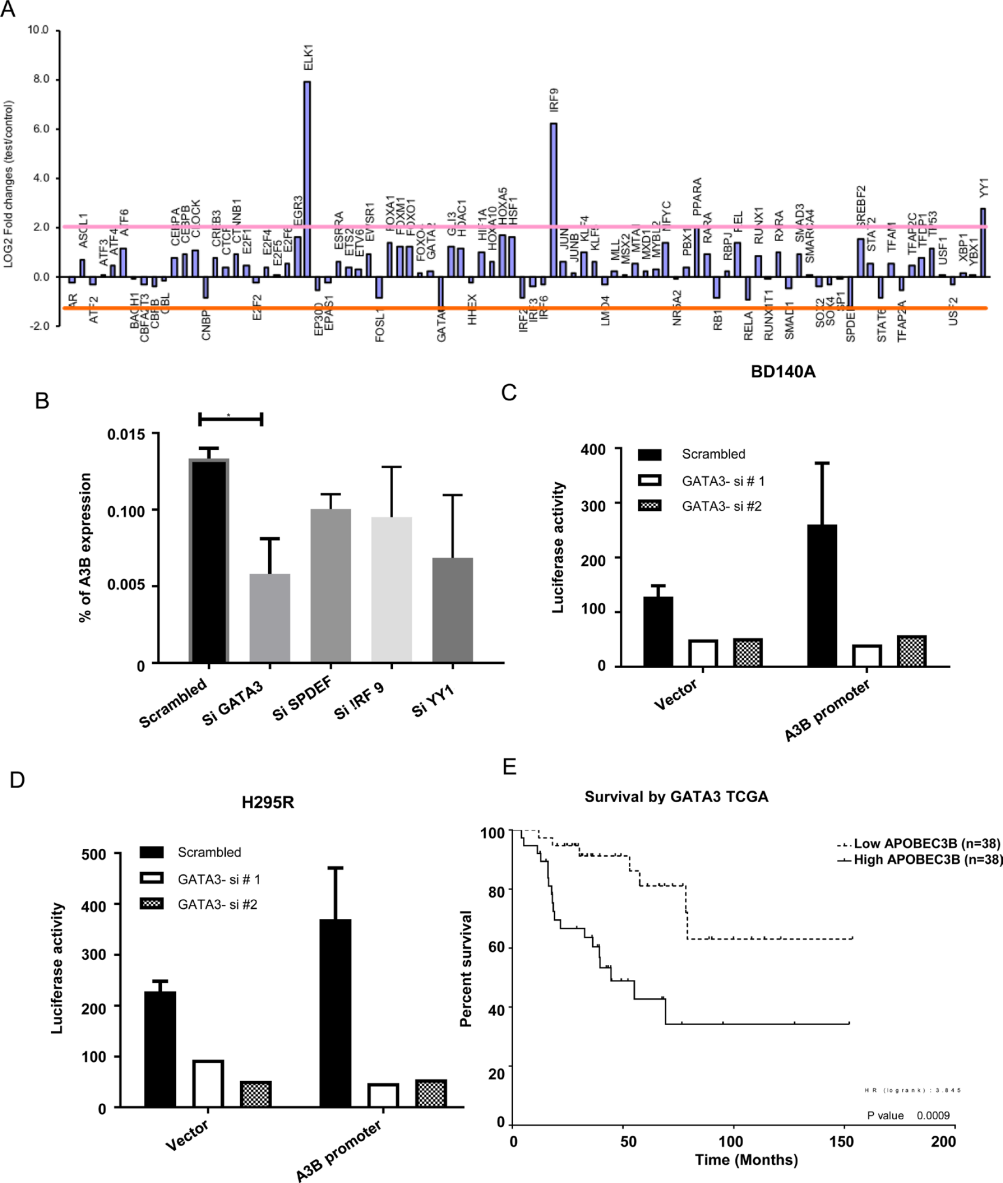
APOBEC3B gene expression is regulated by GATA3. (**A**) APOBEC3B gene expression (log2 fold change) analyzed in a functional screen of 92 cancer-associated transcription factors. (**B**) Independent validation of four candidates by TaqMan gene expression in H295R cells. Luciferase reporter assay under the control of APOBEC3B gene promoter after transient knockdown of GATA3 transcription factor with two different siRNAs in BD140A (**C**) and H295R (**D**) cell lines. (**E**) Overall patient survival dichotomized based on the median gene expression level of GATA3 from the TCGA adrenocortical cancer dataset.

In order to decipher the mechanism by which APOBEC3B gene expression is upregulated in ACC, we analyzed copy number changes, microRNA and CpG methylation data in the same ACC samples that were available from our prior studies [13]. We found no significant copy number differences or differentially expressed microRNA that target *APOBEC3B* and no differential CpG methylation in the *APOBEC3B* promoter or enhancer regions. Therefore, we performed a functional knockdown screen of 92 cancer associated transcription factors and identified GATA3, SPDEF, IRF9, and YY1 as candidate transcriptional activators/repressors for APOBEC3B gene expression (Figure 5A). To validate the screening of the transcription factors, we performed independent siRNA knockdown of the transcription factors and found that APOBEC3B expression was regulated by GATA3 but not by SPDEF, IRF9, and YY1 in H295R cells (Figure 5B). Additionally, we observed a significant positive correlation between GATA3 and APOBEC3B gene expression in two datasets (Supplementary Figure 7). Therefore, we focused on delineating the effect of GATA3 on the transcription of the *APOBEC3B* gene. We carried out a luciferase reporter assay under the APOBEC3B gene promoter after transient knockdown of GATA3 transcription factor with two different siRNAs in BD140 and H295R cells. We found a significant decrease in the APOBEC3B promoter activity when compared to the vector control upon knockdown of GATA3 in both cell lines (Figure 5C and 5D). On further analysis of overall patient survival and disease free survival based on the median gene expression level of GATA3 in the TCGA ACC database, we found a higher overall survival rate in patients with a lower GATA3 expression (*p* = 0.00000) when compared to the patients with a higher expression of GATA3. (Figure 5E, Supplementary Figure 6, and Tables 1 and 2). These results suggest that GATA3 has a critical role in the transcriptional regulation of APOBEC3B gene expression in ACC and an inverse association with patient survival.

**Table 1:** APOBEC3B TCGA OS multivariate analysis

Factor	HR	CI (95%)	*p*-value
**APOBEC3B**			
** Low**	Ref		
** High**	4.325	1.620–11.551	0.003
**Chemotherapy**			
** No chemotherapy**	Ref		
** Chemotherapy**	3.277	0.665–16.145	0.145
**Stage**			
** Stage I**	Ref		
** Stage II**	2.591	0.310–21.633	0.379
** Stage III**	4.418	0.497–39.252	0.183
** Stage IV**	12.664	1.476–108.672	0.021

**Table 2:** GATA3 TCGA OS multivariate analysis

Factor	HR	CI (95%)	*p*-value
**GATA3**			
** Low**	Ref		
** High**	4.898	1.368–17.541	0.015
**Chemotherapy**			
** No chemotherapy**	Ref		
** Chemotherapy**	4.120	0.841–20.180	0.081
**Stage**			
** Stage I**	Ref		
** Stage II**	7.628	0.764–76.189	0.084
** Stage III**	8.321	0.933–74.194	0.058
** Stage IV**	17.369	2.002–150.669	0.010

**Figure 6:**
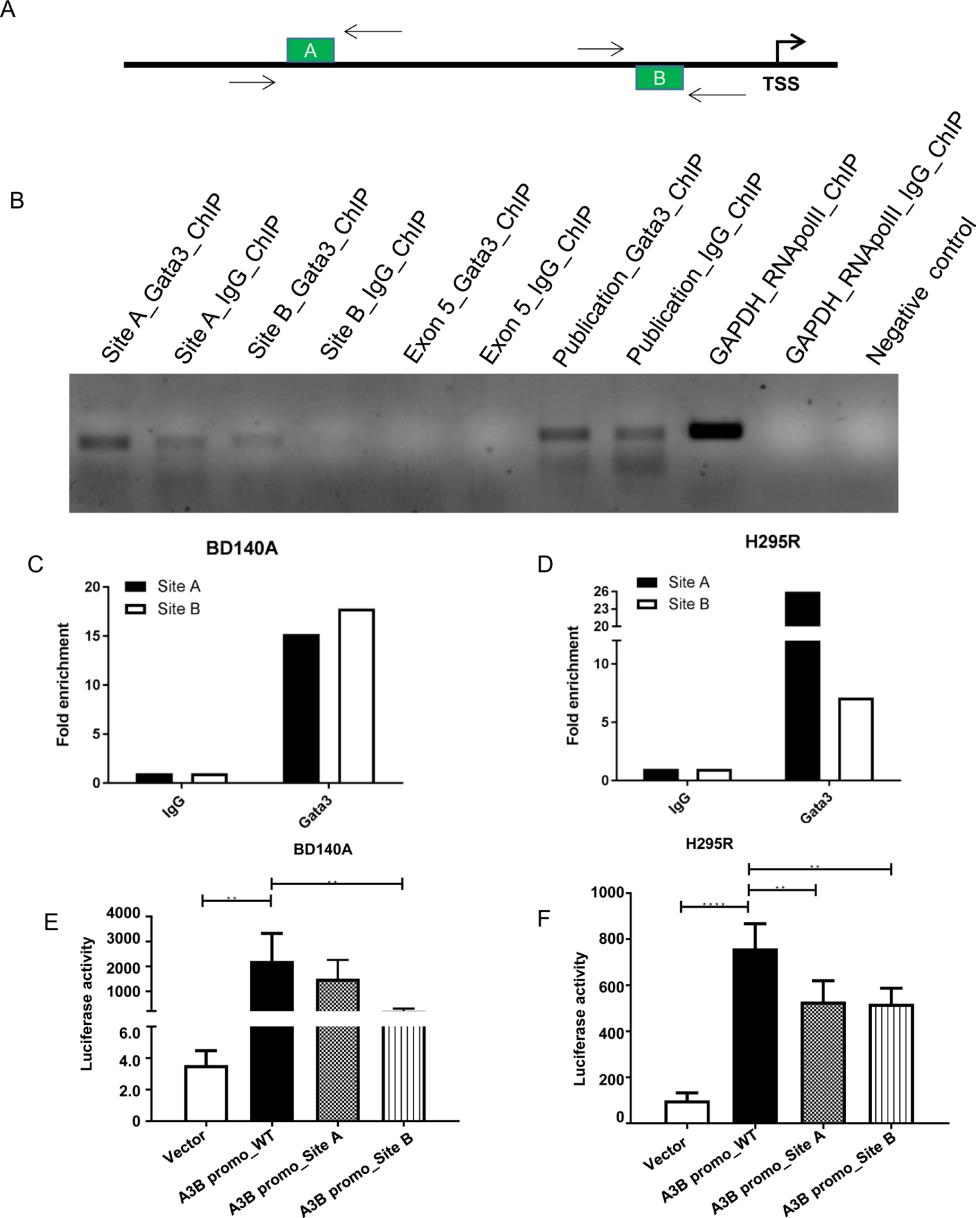
GATA3 binds directly to the APOBEC3B promoter region and regulate its transcription. (**A**) Schematic representation of the GATA3 binding sites on the APOBEC3B promoter region. Arrows indicate the primer probes used for ChiP-PCR; TSS, transcription start site. (**B**) Agarose gel electrophoresis of SYBR green PCR products produced from site A and site B specific primer probes of APOBEC3B promoter region after chromatin immunoprecipitation (ChIP) with GATA3 antibody. Primers were also designed for Exon 5 of APOBEC3B where there are no putative binding sites for GATA3 as a negative control. RNApolII was used as a positive control for ChiP and housekeeping gene GAPDH was used for validation. Previously published GATA3 binding target, GCMB gene specific primers was used as a positive control for GATA3 ChiP antibody [14]. A reaction mixture without template was used as a negative control for PCR. Fold enrichment of GATA3 binding sites A and B after ChIP with GATA3 antibody relative to immunoglobulin (IgG) antibody in BD140A (**C**) and H295R (**D**) cell lines. Luciferase reporter assay after transiently transfecting BD140A (**E**) and H295R (**F**) cell lines with wild-type, site A (G644C) and site B (C910G) mutants in the APOBEC3B promoter region.

Binding of GATA3 to its specific DNA elements in the promoter region of the APOBEC3B gene was determined by ChIP assays. The bioinformatic binding region analysis showed the existence of a GATA3-specific binding element (A, B) in the 5′-promoter region (-280 to -276 bp and -1387 to -1383 bp; + ve strand) of the *APOBEC3B* gene as shown in Figure 6A. Binding of GATA3 to this region was checked at these positions using specific primers. ChIPed chromatin against GATA3 and IgG was de-crosslinked and PCR amplified using the primers for site A and B and positive control primers for GATA3 [14] housekeeping gene GAPDH and RNApolII binding regions, we found higher amplification at site A and B for GATA3 when compared to IgG. The Exon 5 region of APOBEC3B was used as a negative control as it lacks a GATA3 binding site and it showed no PCR amplification, similar to non-template control (Figure 6B). The quantification plot shows a 15-fold enrichment at site A and B in BD140 cell lines, as well as in H295R cells. GATA3 occupancy was 18-fold higher at site A when compared to site B of the APOBEC3B promoter region (Figure 6C and 6D). The functional significance of GATA3 binding at site A and B in the transcriptional regulation of APOBEC3B was validated by using a wild type luciferase reporter assay and mutant A and B APOBEC3B promoter regions. The quantification plot of luciferase activity showed a decrease in the promoter activity of APOBEC3B if the GATA3 binding site A and B were mutated in both BD140 and H295R cell lines (Figure 6E and 6F, and Supplementary Figure 8). These results show the functional relevance of the GATA3 binding site A and B and the regulation of APOBEC3B gene expression in ACC.

### Knockdown of APOBEC3B reduces tumor growth in an ACC xenograft mouse model

**Figure 7:**
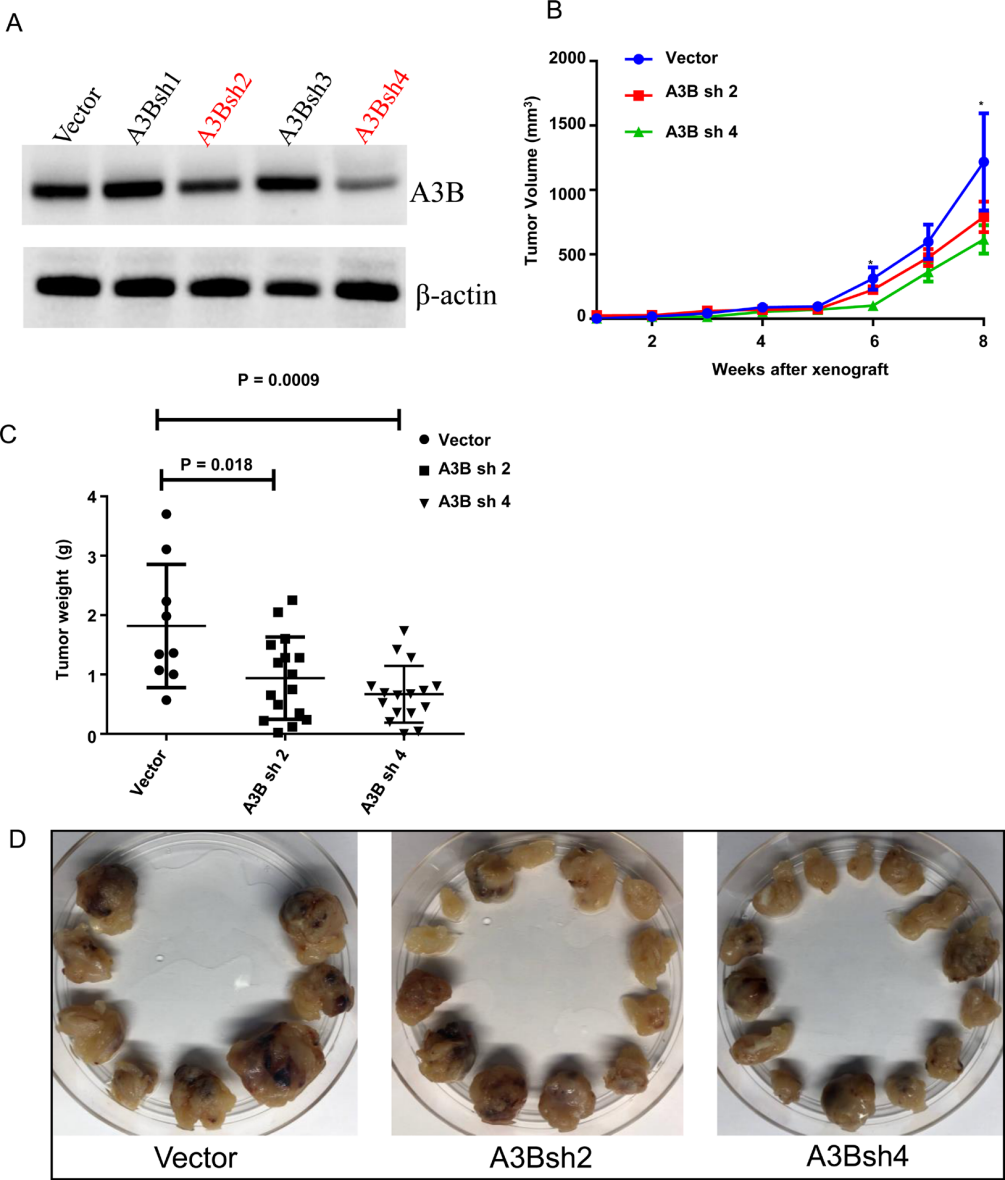
Knockdown of APOBEC3B is associated with a lower tumor growth in an adrenocortical carcinoma xenograft mouse model. (**A**) APOBEC3B protein expression in H295R cells stably transfected with four different shRNAs targeting APOBEC3B. shRNA 2 and shRNA 4 were selected for *in vivo* experiments based on the efficiency. (**B**) Tumor growth curve in NOD scid gamma (NSG) mice that received a vector control, APOBEC3B shRNA2, and 4 stably transfected H295R cells flanks on either side. A total of eight mice per each group were used for this study. Three of the control mice died in the first week after injection for unrelated reasons. The error bars represent the standard error mean (SEM). ^*^Indicates *p* < 0.05. (**C**) The tumor weight of each group at the time of sacrifice (8 weeks after xenograft). (**D**) The isolated tumors from each group at the time of sacrifice.

To examine the function of APOBEC3B *in vivo*, we performed xenograft studies using an immunodeficient mouse model: NOD Scid gamma (NSG) mice. First, we established H295R cell lines that were stably overexpressing empty vector or four different shRNAs (sh1–4) targeting APOBEC3B transcripts. We selected two H295R cell lines (A3B_sh2 and A3B_sh4) that demonstrated a higher knockdown efficiency when compared to the other two, shRNA1 and shRNA3 (Figure 7A). Mice were randomized into three groups with 8 mice in each group. Stably overexpressing vector control, A3B_sh2 and A3B_sh4 were injected subcutaneously into both flanks of each mice. The tumor volume was monitored every week for up to 8 weeks. We found a significant difference in tumor volume between the empty vector group and the APOBEC3B shRNA groups at week 6 and week 8 (Figure 7B). The tumor weight was significantly lower in A3Bsh2 (*p* = 0.018) and A3Bsh4 (*p* = 0.0009) as compared to the empty vector control (Figure 7C and 7D).

## DISCUSSION

Here we report for the first time that APOBEC3B is overexpressed in ACC as compared to benign adrenocortical tumors and normal adrenal cortex tissues and this is associated with a higher copy number alteration, higher rate of TP53 mutations and DNA damage in ACC. Functional studies of APOBEC3B revealed that it has an effect on cellular growth, both *in vitro* and *in vivo*. Previous studies have shown an association between APOBEC3B expression level and aggressive clinicopathologic characteristics and its expression is highly correlated with cellular proliferation in breast cancer [15]. In addition, APOBEC3B knock down in ovarian cancer cells [12] and glioma [16] results in decreased cell viability and proliferation. We show that GATA3 directly binds to the promoter region of APOBEC3B and transcriptionally regulates its gene expression in ACC. Lastly, we show that both the expression level of APOBEC3B and GATA3 are prognostic markers in patients with ACC.

Recent studies have reported that APOBEC3B-associated mutagenic agents have an effect on the evolution of various cancer types and it is significantly overexpressed in a variety of cancers (such as breast, gastric, lung, and ovarian) compared to corresponding normal tissues [8, 17, 18]. In our study, we also found that APOBEC3B was overexpressed in ACC. However, the role of APOBEC3B beyond its involvement in inducing DNA damage [19–21], DNA replication [22] and proliferation has not been well-characterized in the context of any cancer type. Transient functional knockdown studies in two ACC cell lines significantly reduced cellular proliferation, suggesting that APOBEC3B may have a growth-promoting function in ACC, and knockdown of APOBEC3B gene expression increased the number of cells in S-phase arrest.

The members of the APOBEC3 family possess the ability to induce detectable DNA damage and activation of the DNA repair machinery as associated with strong pan-nuclear γ-H2AX signal [18, 23–25]. In our study, the higher levels of γ-H2AX were detected in ACC as compared to normal adrenal cortices and benign adrenocortical tumors with partial co-localization within the nucleus of APOBEC3B.

The *TP53* gene is frequently mutated in many cancers and tumors with *TP53* mutations have also been reported to have higher levels of APOBEC3B expression in breast cancer [26] and ovarian cancers [12]. Our results show that APOBEC3B expression level is associated with a higher rate of *TP53* mutations in ACC [12, 18]. We found *TP53* mutations in the intron of the gene which have been found to alter TP53 protein expression through several mechanisms such as aberrant pre-messenger mRNA splicing and disruption of DNA-transcription factor interactions in a variety of cancer types, as well as, single nucleotide polymorphism in introns associated with higher cancer risk [27–29]. This suggests that overexpression of APOBEC3B might contribute to ACC progression.

Several studies looking at ACC have identified molecular events that may be responsible for disease progression [30]. Zhao *et al*. reported gains in the DNA copy number in chromosome 17 or 17q to represent the earliest genomic alterations which are seen in benign adrenocortical tumors [30]. A study by Dohna and colleagues, reported gains and high-level amplifications in chromosomes 7, 14, and 19 in ACC but not in benign adrenocortical neoplasms, suggesting that these events may be late genetic alterations during tumorigenesis [31]. In our cohort, we found that ACC tumor samples with a high APOBEC3B expression had a higher number of chromosomal gain/loss particularly in chromosome 4 and 8 as compared to tumor samples with a low APOBEC3B expression, suggesting that it may have a role in chromosomal instability. The two datasets where we did not see any significant correlation may be due to the small sample size.

Several reports showed that higher levels of APOBEC3B expression was associated with decreased survival rates among estrogen receptor-positive (ER+) breast cancer patients [32] and a higher expression of APOBEC3B was independently associated with lower survival rates of ovarian cancer patients [12]. Our analysis using the TCGA ACC database also showed a significant association with lower survival rate in patients with higher APOBEC3B expression. However, we did not see a significant association between APOBEC3B expression and overall survival in our cohort and other public datasets but the sample sizes were small.

Very little is known about the mechanism by which APOBEC3B gene expression is regulated. Several genomic studies have revealed that there was no evidence of copy number alterations, presence of miRNA binding sites and methylation CpG sites in the genomic region of APOBEC3B suggesting that it is not regulated by any of the globally known mechanisms [18]. We also found no dysregulated microRNA targeting APOBEC3B and differential CpG methylation in the gene promoter and enhancer regions or copy number alterations in the gene using our previous genomic data in the same ACC sample sets. Based on the functional screening of 90 cancer-associated transcription factors, we found that GATA3 is a positive regulation of *APOBEC3B* gene expression. In breast cancer, a low expression of GATA3 has been reported to be strongly associated with a higher histologic grade, poor differentiation, positive lymph nodes, Estrogen Receptor (ER) and progesterone receptor (PR) negative and overexpressed HER2/neu, all of which are indicators of poor prognosis [33]. GATA3 levels are also inversely associated with the metastatic capability of various human breast cancer cell lines [34]. In ACC, GATA3 is weakly expressed [35] and through the analysis of the TCGA ACC database, we found an association between GATA3 expression and patient survival, which was higher in patients with a low level of GATA3 expression.

In summary, APOBEC3B overexpressed in ACC, and is associated with DNA damage, S phase arrest, higher copy number alterations and *TP53* mutations in ACC. For the first time, we demonstrated that GATA3 directly regulates the expression of APOBEC3B and that both are prognostic markers in ACC.

## MATERIALS AND METHODS

### Patient samples

Human adrenocortical tissue samples were collected according to an institutional review board-approved clinical protocol after written informed consent was obtained (NCT01005654 and NCT01348698).

### Cell culture

Human ACC cell lines H295R and SW13 were purchased from ATTC (Rockville, MD, USA) and were grown in DMEM supplemented with 1% insulin transferrin selenium and 2.5% NuSerum I (Corning, Bedford, MA, USA). The ACC BD140A cell line [13, 36, 37] was kindly provided by Drs. Kimberly Bussey and Michael Demeure (TGen, Pheonix, AZ, USA), it was cultured in RPMI-1640 medium supplemented with 10% FBS, 1% penicillin-streptomycin and 1% L-glutamate (Life Technologies, Grand Island, NY, USA). Human embryonic kidney cell line HEK293 was cultured in DMEM supplemented with 10% FBS (Sigma-Aldrich) and 1% penicillin-streptomycin. All the cancer cell lines were authenticated by short tandem repeat profiling and had testing for mycoplasma contamination. All experiments were done with cells from the original stock with no more than 15 passages.

### Gene expression analysis from public datasets

The microarray data from the GEO database, GSE10927 was analyzed through an online GEO2R expression analysis software using the default parameters and the APOBEC3B gene expression values for each patient samples were collected and plotted in Box and whiskers graph. The microarray gene expression data from EMBL-EBI, E-TABM-311 dataset was directly extracted from the author submitted normalized expression data and all the data points for APOBEC3B gene probe were shown in Box-whiskers plots.

### Cell transfection and Luciferase reporter assays

For transient transfection, two siRNAs targeting APOBEC3B (A3Bsi#1, s18411 and #2, s18412), two siRNAs targeting GATA3 (Gata3si#1, s5599, and si#2, s5600) and a negative control (Scrambled); Life Technologies, Thermo Fisher Scientific), were transiently transfected into 2 × 10^5^ cells in 6-well plates, using the transfection reagent RNAiMax (Invitrogen, Thermo Fisher Scientific) according to the manufacturer’s protocol. Four lentiviral psi-LVRU6P-01, psi-LVRU6P-02, psi-LVRU6P-03 and psi-LVRU6P-04 vectors (catalog # CS-HSH022939-LVRU6P-01 to LVRU6P-04) with U6 promoter expressing shRNAs targeting APOBEC3B and one shRNA scrambled control clone for psi-LVRU6P (catalog # CSHCTR001-LVRU6P) were obtained from GeneCopoeia (Rockville, MD, USA). H295R cells were transfected with four shRNAs and vector control using Lipofectamine 2000 and selected with 1 μg/ml puromycin for one month. Stable H295R cells expressing shRNAs 2 and 4 were used for xenograft studies as they have shown high knockdown efficiency. For luciferase reporter experiments, the cells were seeded into a 96-well plate (15,000 cells per well). After 24 hours, the cells were co-transfected with a GoClone reporter vector containing the promoter region of APOBEC3B (SwitchGear Genomics, Active Motif) or mutant vectors and the siRNAs targeting GATA3 (Applied Biosystems, Thermo Fisher Scientific), using the Lipofectamine 2000 reagent (Invitrogen) according to the manufacturer’s protocol. Luciferase activity was measured 24 hours after transfection, using the LightSwitch Luciferase Assay Reagent (SwitchGear Genomics) according to the manufacturer’s protocol.

### Chromatin immunoprecipitation assay

Chromatin immunoprecipitation (ChIP) assays were performed using the Chromatin Immunoprecipitation Assay Kit (Magna ChIP; Millipore) according to the manufacturer’s instructions. Briefly, the cells were cross-linked by 1% formaldehyde for 10 minutes. The formaldehyde was quenched using 2 ml of 10× glycine for 5 minutes at room temperature before harvesting. Cells were collected by centrifugation in phosphate-buffered saline (PBS) containing protease inhibitors and were lysed in SDS lysis buffer. Soluble chromatin was prepared after sonication to a DNA length of 200 to 500 base pairs. Fragmented chromatin was immunoprecipitated using antibodies against GATA3 (Abcam) or IgG control overnight at 4°C on a rotating platform. The magnetic beads were washed, the chromatin was extracted and protein-DNA complexes were reverse cross-linked. DNA was purified and analyzed by PCR using the specific primers for the APOBEC3B promoter. The total input was the supernatant from the no-antibody control. The following ChiP primers were used for site A (for: CTCCCAGGCTCAGGCTGC and rev: CTGGTTTTCCCCGGACCCT), site B (for: AGGAAGTGAAACCACAGA and rev: GACCACCAGGCAGGAAGG), Exon 5 as negative control (for: CTGGGAGGACAGGCCAG and rev: TTTTCATCGAATTTGTAC), GCMB gene as a positive control from literature [14]. (for: AAGATTTGTGTTCGGGTGGG and rev: TGGGGAAGGAGGGAAAGGGG) and GAPDH positive control (for: TACTAGCGGTTTTACGGGCG and rev: TCGAACAGGAGGAGCAGAGAGCGA).

### RNA extraction and real-time PCR

Total RNA was extracted from snap-frozen tissues and cell lines using TRIzol reagent (Invitrogen, Thermo Fisher Scientific) according to the manufacturer’s protocol. RNA yield was determined using a NanoDrop 2000 spectrophotometer (Thermo Fisher Scientific).

Gene expression levels were measured using specific primers and probes. Briefly, 500 to 1,000 ng of total RNA was reverse transcribed using a High Capacity Reverse Transcription cDNA kit (Cat #4374967, Applied Biosystems, Thermo Fisher Scientific) and the resulting cDNA was diluted and amplified according to the manufacturer’s instructions. The taqman probes were purchased from Thermofisher scientific. For human APOBEC3B (ID#Hs00358981_m1), human GATA3 (ID#Hs00231122_m1) and human GAPDH, ID#Hs02786624_g1). GAPDH was used as an endogenous control [38–41]. Gene expression levels were calculated using SDS 2.3 software (Applied Biosystems, Thermo Fisher Scientific). The percent gene expression was calculated as the normalized gene expression level = 2 ^– (C^t ^of gene of interest – C^t ^of GAPDH)^ × 100%, where C_t_ is the PCR cycle threshold. All PCR reactions were performed in a final volume of 20 μl with 1 μl of cDNA template on an ABI PRISM^®^7900 Sequence Detection System (Applied Biosystems). The PCR thermal cycler condition was 95°C for 12 minutes followed by 40 cycles of 95°C for 15 seconds and 60°C for 1 minute. All experiments were performed in triplicate.

### Cell proliferation assay

Cell proliferation experiments were performed using CyQUANT cell proliferation assays (Life Technologies) according to the manufacturer’s protocol. For transiently transfected cells, the cells were first transfected in a six-well plate, trypsinized 24 hours later, counted and then seeded in quadruplicate on ninety-six-well black plates to monitor cell growth. Absorbance at 485 nm/538 nm was determined using a ninety-six-well fluorescence microplate reader SpectraMax M5 (Molecular Devices, Sunnyvale, CA, USA). H295R cells were seeded at 8,000 cells/well and BD140A cells at 4,000 cells/well.

### Cell cycle assay

Cells were reverse transfected with control and APOBEC3B specific siRNAs, plated at a density of 2 × 10^5^ cells in a 6 well dish with 2 mL of culture medium. Three days later, cells were trypsinized, washed with PBS and fixed with ice-cold 70% ethanol. Cells were then washed with PBS and resuspended in a solution consisting of PBS, ribonuclease A (0.1 mg/mL) and propidium iodide (0.05 mg/mL). The DNA content of cells was then measured by FACS analysis using Canto II (Becton Dickinson). Cell cycle analysis of the gated propidium iodide (PI) distribution was done using ModFit software (Verity Software House, Inc.).

### Immunofluorescence staining

Tissues were fixed with 10% formalin and embedded in paraffin. Tissue-section slides were deparaffinized in xylene and rehydrated in ethanol, followed by antigen retrieval with 10% citrate buffer (pH 6.0) in a pressure cooker for 10 minutes at 120°C. Slides were incubated with the primary antibody at 4°C overnight. For APOBEC3B and ɣH2AX staining, Anti-APOBEC3B antibody (Abcam, Cambridge, MA, ab222330) and Anti-gamma H2AX (phospho S140) antibody (ab22551) was used at 1:200 dilution. The rabbit and mouse secondary antibodies were used from Invitrogen Alexa fluorescently conjugated system.

### Functional screening of transcription factors

SureFIND transcriptome PCR array cancer associated transcription factor assay was purchased directly from SABiosciences (now Qiagen Inc, catalog # TCSC-401C-1), (Germantown, MD, USA). Briefly, MCF7, breast cancer cells were transiently knockdown with a pool of siRNAs targeting each of the 92 cancer associated transcription factors independently and the RNA was extracted and subsequent cDNA was generated from these cells and aliquoted onto a 96-well PCR plate. The APOBEC3B gene expression was then assayed and the log2 expression values were calculated with reference to the house keeping gene, GAPDH, according to the manufacturer’s recommendation and their provided template.

### Site-directed mutagenesis

The mutations in the APOBEC3B promoter constructs were generated by site-directed mutagenesis of a wild-type (WT) human APOBEC3B promoter clone obtained from Switchgear genomics using QuickChange Site-directed mutagenesis kit from Stratagene (Santa Clara, CA, USA). The following primers were used for generating the mutant constructs for site A (for: CCTGCCCAGGACACATAAAGACAGAGCAGC and rev: GCTGCTCTGTCTTTATGTGTC CTGGGCAGG) and site B (for: CAGGGACAAGCGTATGTAAGAGGCTGAACATG and rev: CATGTTCAGCCTCTTACATACGCTTGTCCCTG). The numbering of the position was made based on the promoter region of the clone obtained from Switchgear genomics.

### TP53 gene sequencing

The exon and interon regions of *TP53* were sequenced at the NCI Sequencing Facility (Frederick, MD, USA).

### Comparative genomic hybridization array analysis

For the SNP-CGH array experiment, Illumina HumanCytoSNP-12v2.1 BeadChip was used. Data generated were then analyzed by using the ASCAT method described by Van Loo *et al*. [42]. We compared and schematically represented the copy number status and clustering of the patients in our cohort using Nexus copynumber software (BioDiscovery, Inc, El Segundo, CA, USA).

### Protein extraction and western blots

Cells were lysed in a buffer containing 10 mM Tris and 1% SDS with 1× Halt Protease and a phosphatase inhibitor cocktail (Thermo Fisher Scientific) was used. The lysates were quantified for protein concentrations using a BCA Protein Assay kit (Pierce, Life Technologies). Cell lysates were analyzed in SDS-PAGE and transferred onto polyvinylidene difluoride (PVDF) membranes (Invitrogen, Thermo Fisher Scientific). The membranes were blocked with 5% BSA in a TBS-Tween buffer and then incubated with primary antibodies overnight at 40°C. The primary antibodies used were: APOBEC3B antibody (1:1000, Abcam, ab191695); β-actin antibody (1: 5000, Santa Cruz Biotechnology, sc-47778), phospho-p44/42 MAPK (Erk1/2) (Thr202/Tyr204) antibody (1:1000, Cell Signaling, #4370) and p44/42 MAPK (Erk1/2) antibody (1:1000, Cell Signaling, #9102). Secondary antibodies used were goat anti-mouse IgG-horseradish peroxidase (HRP) and goat anti-rabbit IgG-HRP (1:5000, Santa Cruz Biotechnology, USA).

### Xenograft studies

NOD. Cg-Prkdcscid Il2rgtm1Wjl/SzJ (NOD scid gamma, NSG) breeder female mice were purchased from The Jackson Laboratory, they were maintained and bred according to the guidelines of the National Institutes of Health Animal Research Advisory Committee (NIH-ARAC). NSG mice were xenografted when they were 3–4 months old. A total of 24 mice, no more than 5 animals in each cage, weighing 27–33 g, were randomly assigned to three groups, each group receiving a subcutaneous injection on both sides of the flank with 3 × 10^6 H295R cells stably expressing either APOBEC3B shRNA2, APOBECB shRNA4 or the control vector in a 100-μl suspension (cell culture medium: Matrigel = 1:1). A blinded measurement of the tumor size was taken each week. Tumor volumes were calculated using the equation ×2y/2, where x is the smallest and y is the largest diameters. Isoflurane was used as an anesthetic agent as it is safer than the other anesthetics. Mice were euthanized primarily by inhalant anesthetics (CO_2_ or isoflurane) for a vaporizer followed by a secondary physical method of cervical dislocation to ensure the animal death as per the NIH animal safety guidelines. Mice were sacrificed when the tumor size reached 2 cm in greatest diameter. The animal experiments were approved by the NIH-ARAC.

### Survival analysis

Overall survival was defined as the time from preoperative blood samples drawn to disease-related mortality or last follow-up, which was censored for those patients who underwent repeated abdominal surgery. The association between APOBEC3B gene expression levels and survival was assessed using the Kaplan–Meier method, with statistical differences determined by log-rank test. *P* < .05 was considered statistically significant. All calculations were performed using GraphPad Software 6.0.

### Statistical analysis

For gene expression data in Figure 1A–1C, we have performed a one way ANOVA with Sidak’s multiple comparisons test. For gene knockdown and cell proliferation experiments in Figure 2A–2D, we have used two way ANOVA statistical testing with Dunnett’s correction for multiple comparison. Error bars in the graphs represent the standard deviation (SD) across the samples whereever applicable.

## SUPPLEMENTARY MATERIALS



## Abbreviations

APOBEC3B: Apolipoprotein B mRNA editing enzyme catalytic subunit 3B
ACC: Adrenocortical carcinoma
ChIP: Chromatin immunoprecipitation
