# Supplementary material for: GATA3 and APOBEC3B are prognostic markers in adrenocortical carcinoma and APOBEC3B is directly transcriptionally regulated by GATA3
Source: Oncotarget. 2020 Sep 8;11(36):3354–70. doi: 10.18632/oncotarget.27703 (PMC7486697; doi:10.18632/oncotarget.27703)
Supplement: Supplementary file 1 [file oncotarget-11-3354-s001.pdf]

GATA3 and APOBEC3B are prognostic markers in adrenocortical carcinoma and APOBEC3B is directly transcriptionally regulated by GATA3

SUPPLEMENTARY MATERIALS

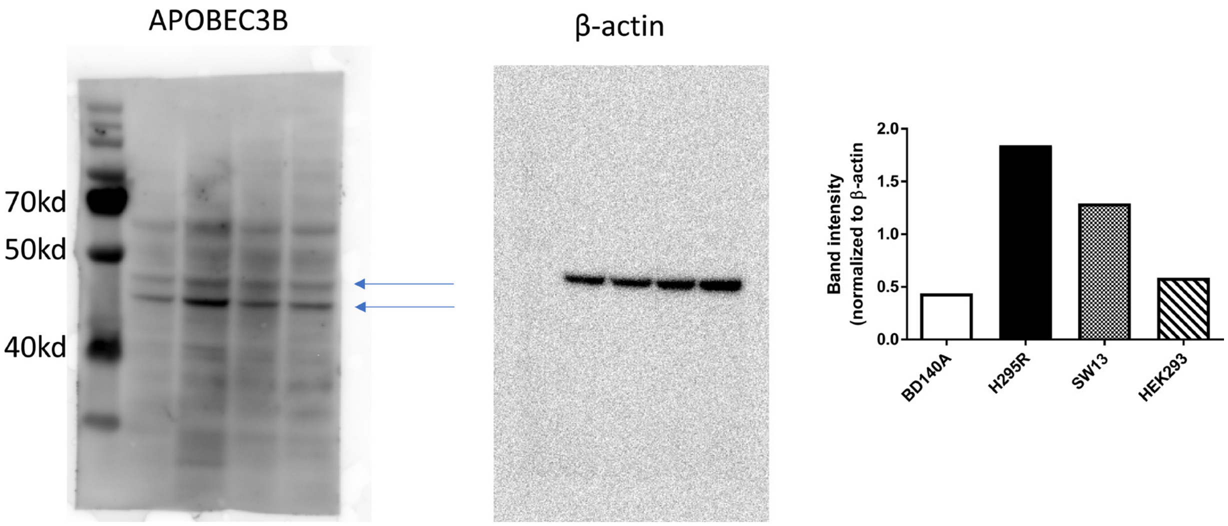

Supplementary Figure 1: Validation of knockdown of APOBEC3b gene and protein expression. Full western blot of APOBEC3B gene and protein expression upon silencing with reference to loading control b-actin.

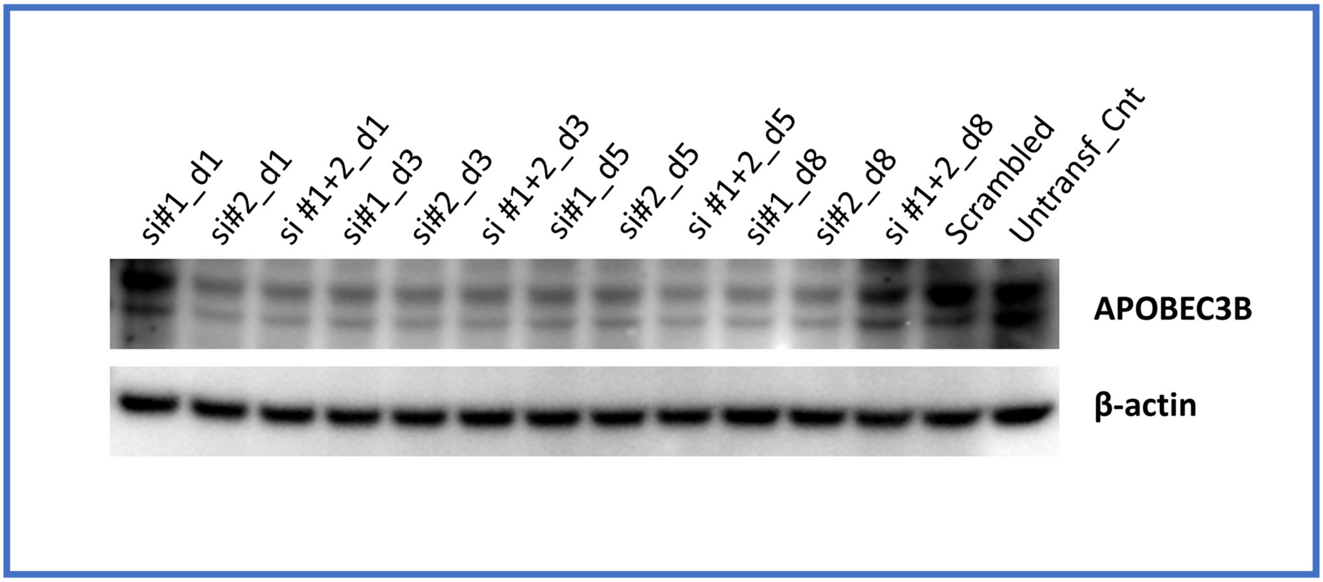

Supplementary Figure 2: Validation of knockdown of APOBEC3B protein expression in H295R cell line. Western blot of APOBEC3B protein expression upon silencing with two independent siRNAs and with reference to the loading control b-actin.

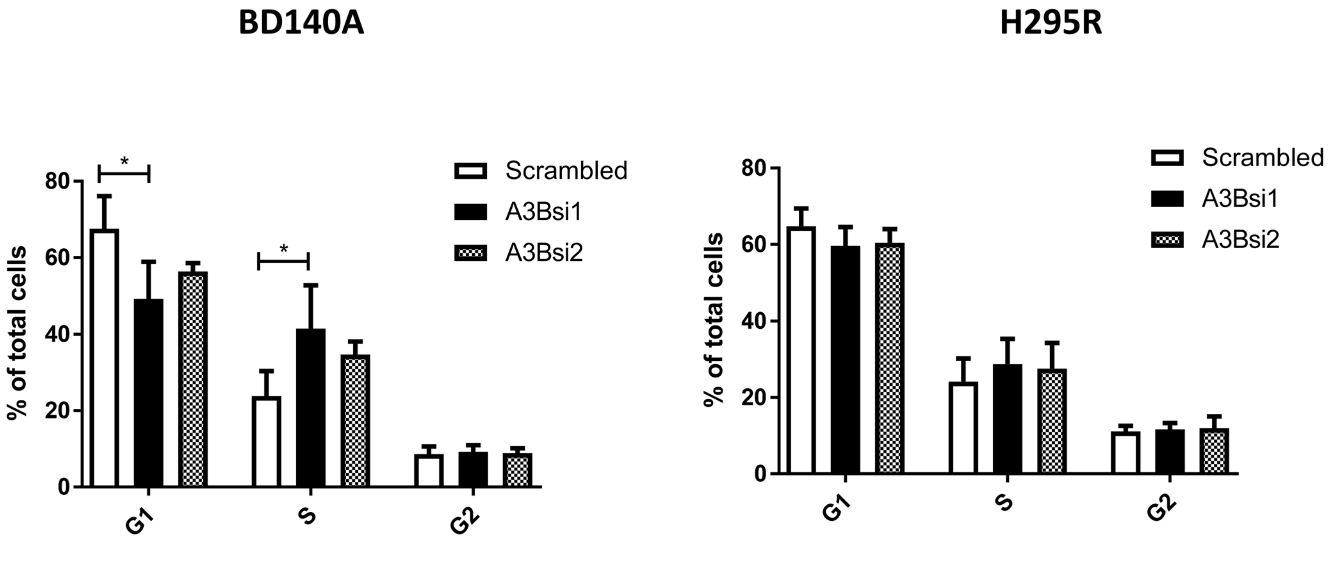

Supplementary Figure 3: Quantification of cell cycle analysis from flow cytometry. Quantification of cell cycle stages (G1, S, and G2 phases) after silencing APOBEC3B gene expression using two independent siRNAs in BD140A and H295R adrenocortical cell lines. Data shown are from two independent experiments and $^{*}p < 0.05$.

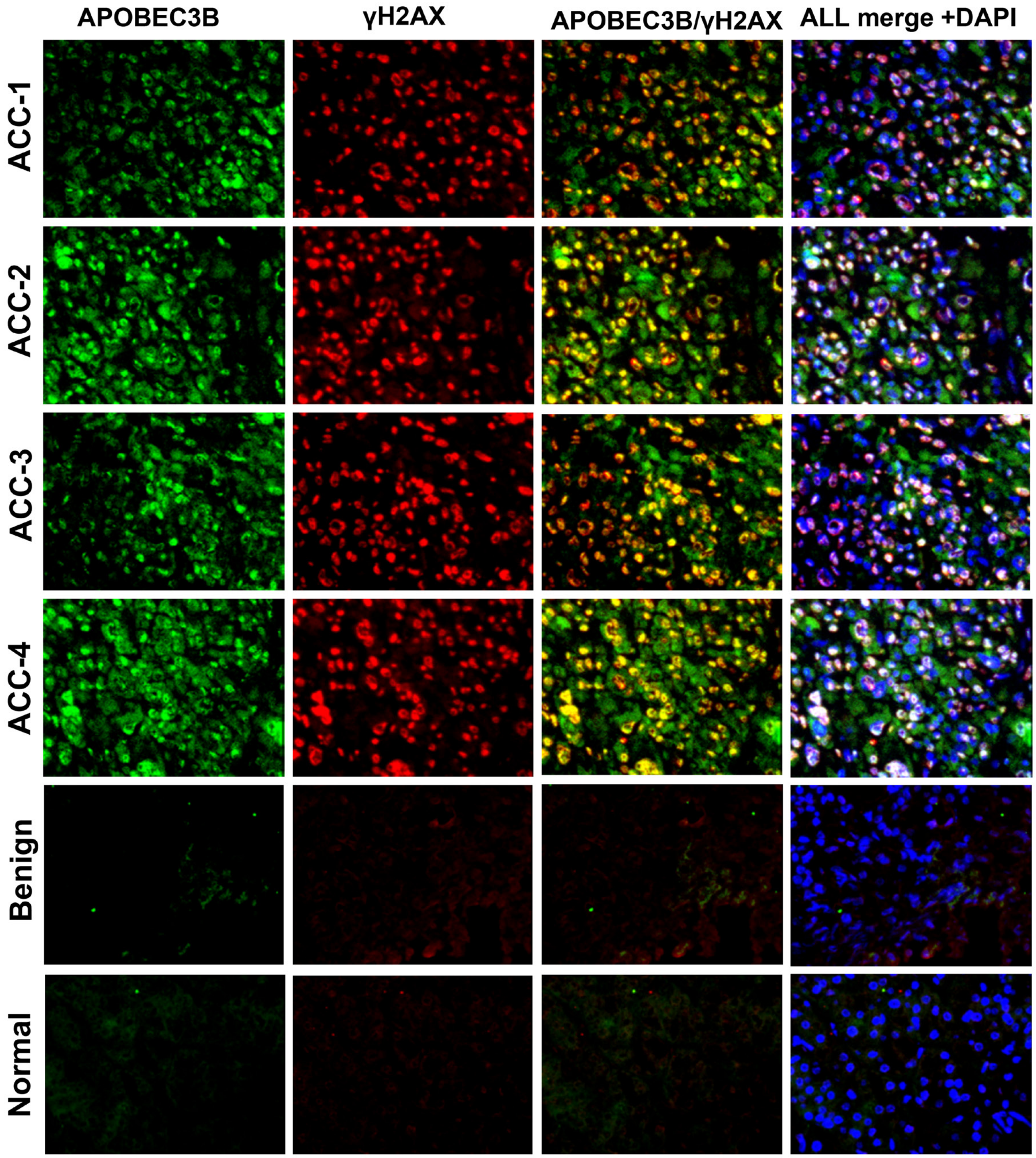

Supplementary Figure 4: Co-localization of APOBEC3B expression with γH2AX: Immunofluorescence staining images of APOBEC3B expression and γH2AX in normal, benign and ACC tumors (magnification – 40×).

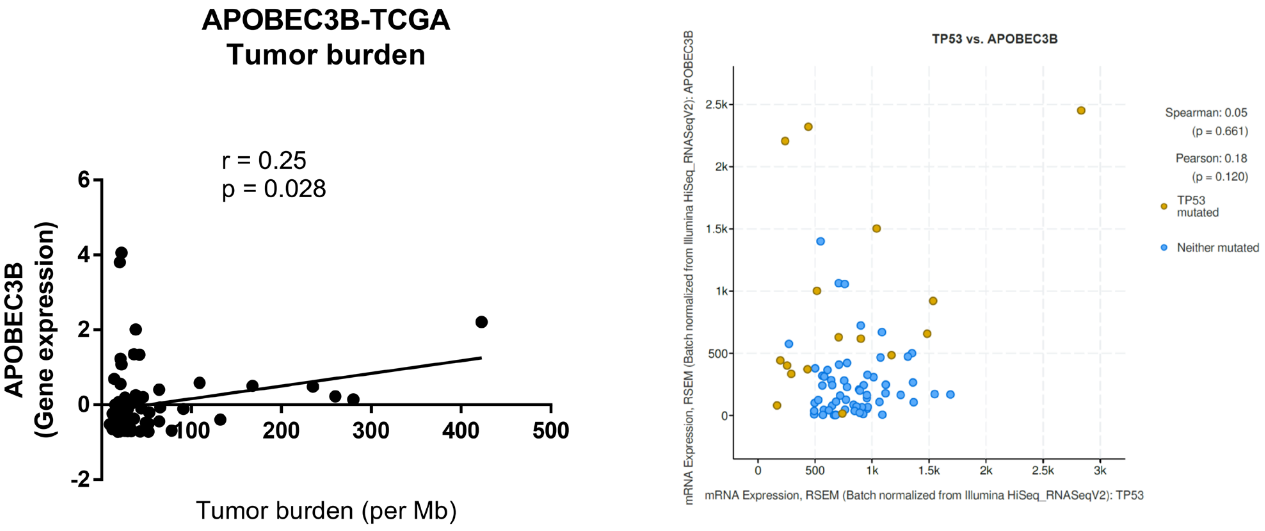

Supplementary Figure 5: APOBEC3B gene expression and mutation burden. Correlation between APOBEC3B gene expression and the overall tumor burden (per Mb) from the TCGA dataset. Co-occurrence of APOBEC3B expression and TP53 mutation rate as a whole in TCGA ACC dataset.

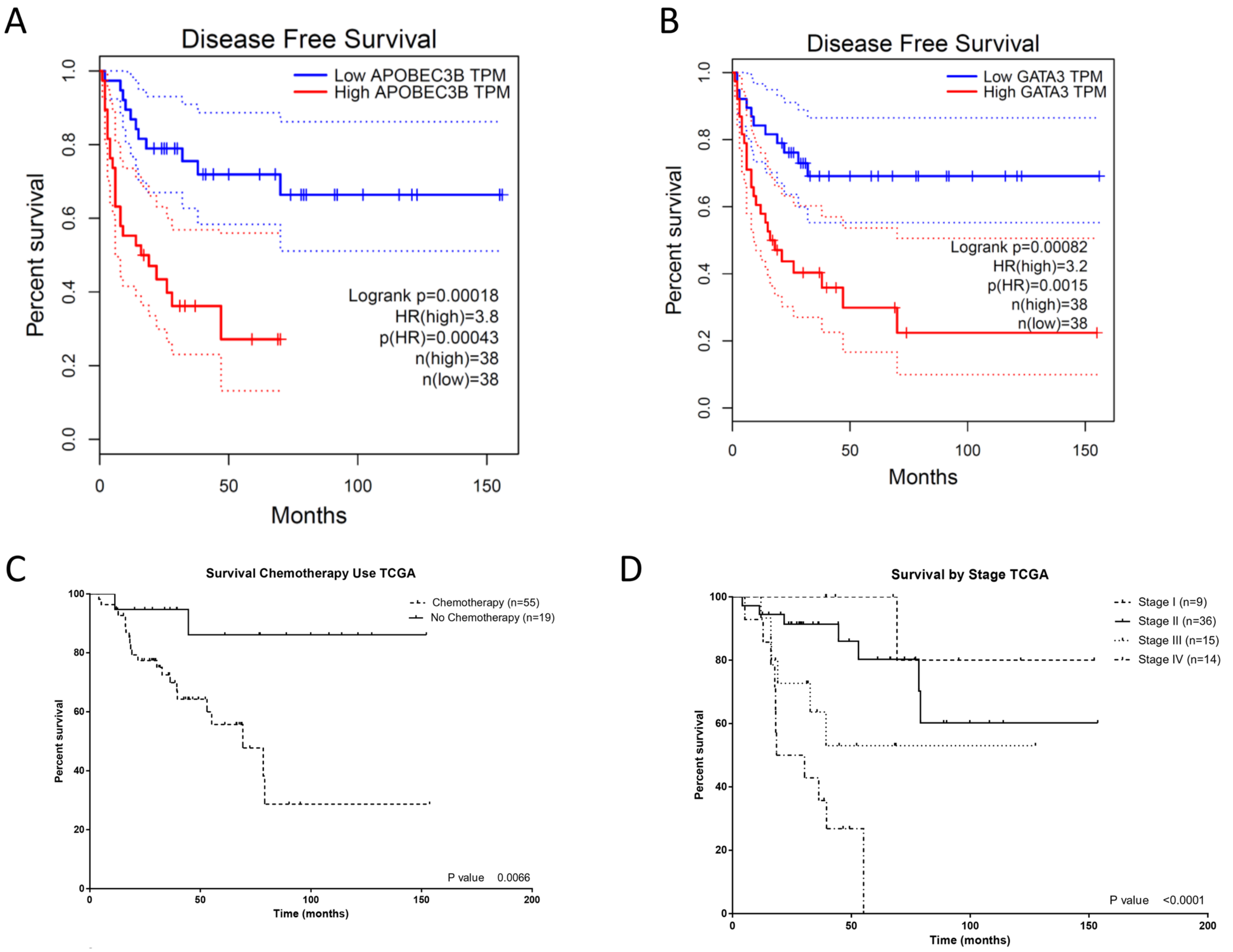

Supplementary Figure 6: Disease free survival of APOBEC3B and GATA3 from TCGA dataset. The disease-free survival was calculated based on the median gene expression of APOBEC3B (**A**) and GATA3 (**B**). The 95% confidence interval was added as a dotted line. Survival analyses based on chemotherapy (**C**) and by stage (**D**) from the TCGA adrenocortical cancer dataset.

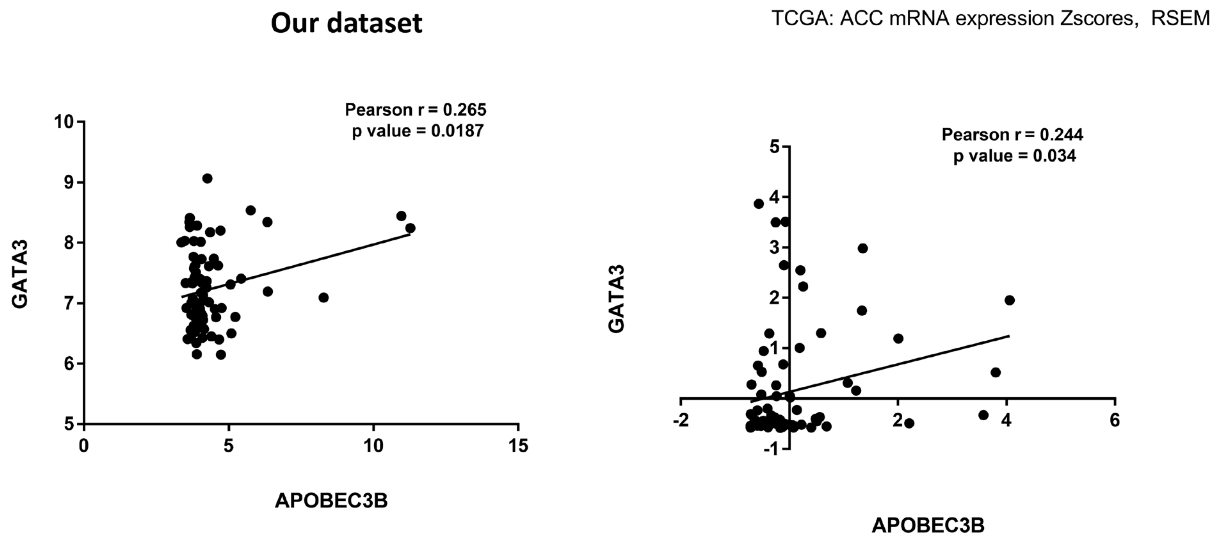

Supplementary Figure 7: Correlation between APOBEC3B and GATA3. Correlation between APOBEC3B and GATA3 gene expression in our dataset and from the TCGA dataset.

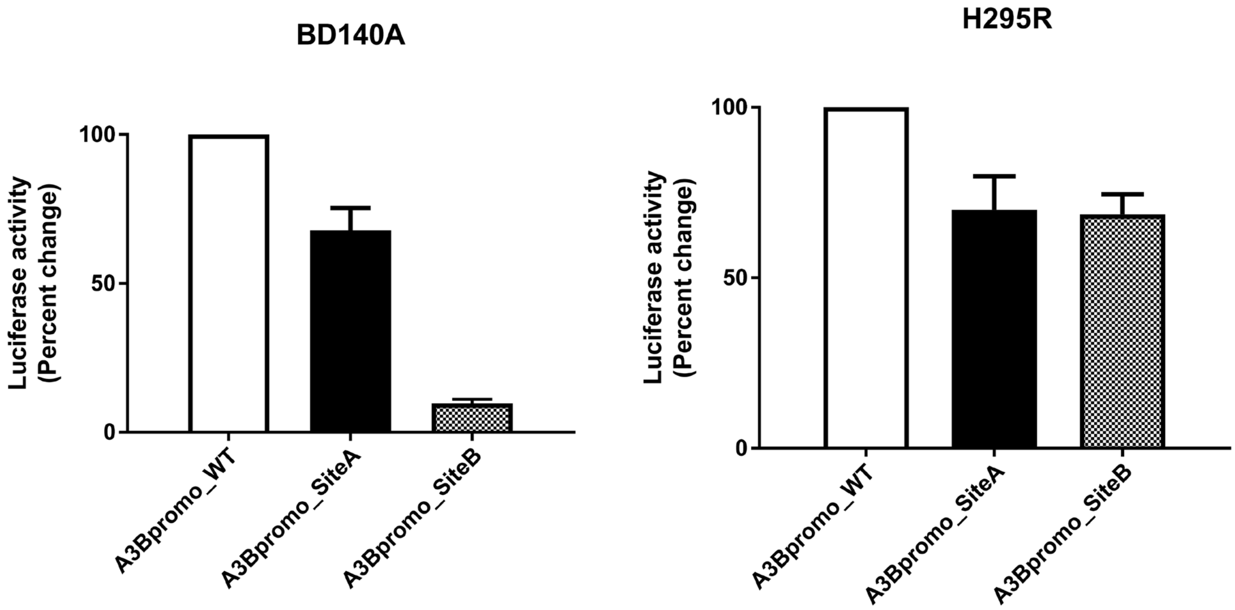

Supplementary Figure 8: Effect of mutations in APOBEC3B promoter region. Percent change of luciferase signal in BD140A and H295R cell lines expressing mutant GATA3 binding sites with in the APOBEC3B promoter region.
